# Association between Participants' Characteristics, Patient-Reported Outcomes, and Clinical Outcomes in Youth with Sickle Cell Disease

**DOI:** 10.1155/2018/8296139

**Published:** 2018-07-18

**Authors:** Sherif M. Badawy, Leonardo Barrera, Stephanie Cai, Alexis A. Thompson

**Affiliations:** ^1^Department of Pediatrics, Northwestern University Feinberg School of Medicine, Chicago, IL, USA; ^2^Division of Hematology, Oncology and Stem Cell Transplant, Ann & Robert H. Lurie Children's Hospital, Chicago, IL, USA; ^3^Northwestern University Feinberg School of Medicine, Chicago, IL, USA

## Abstract

**Background:**

Sickle cell disease (SCD) is a chronic debilitating illness. SCD-related complications result in substantial impairment in quality of life (QOL). Our study objective was to assess the relationship of participants' characteristics, QOL, hydroxyurea adherence, and SCD-related clinical outcomes in youth with SCD.

**Procedure:**

A single-center cross-sectional study. Thirty-four youth with SCD enrolled from clinic between January and December 2015. Participants completed PROMIS® measures and ©Modified Morisky Adherence Scale.

**Results:**

Participants had a mean age of 14.8 (SD 2.9) years and 41% were female. Participants' age correlated with fatigue (r_s_=0.48;* P*=0.006), pain (r_s_=0.32;* P*=0.07), and anxiety (r_s_=0.33;* P*=0.06) scores. Participants with chronic pain had worse upper extremity physical function (*P*=0.05), pain (*P*=0.04), anxiety (*P*=0.05), and depression (*P*=0.05). Males reported significantly higher hydroxyurea adherence (5.4 versus 3.6,* P*=0.02) compared to females. Participants with chronic pain had more frequent hospitalizations (*P*=0.02), emergency room visits (*P*=0.04), and longer total length of hospital stays over 12-month period (*P*=0.01).

**Conclusions:**

Older and female participants had worse QOL scores, and males reported higher hydroxyurea adherence. Participants with chronic pain reported significant impairment in different QOL domains and had increased healthcare utilization. Future longitudinal studies examining the relationship between participants' characteristics, QOL, hydroxyurea adherence, and SCD-related clinical outcomes are needed.

## 1. Introduction

Sickle cell disease (SCD) is a chronic debilitating illness affecting more than 5 million individuals worldwide and about 250,000 live births every year have SCD. [1] In the United States, SCD is seen in about 100,000 Americans, mainly of African American descent. [2] Patients with SCD suffer from the following complications: unexpected pain episodes, chronic anemia, acute chest syndrome, pulmonary hypertension, kidney disease, gall stones, strokes, and long-term end-organ damage. [3] These SCD-related complications result in substantial impairment in quality of life (QOL) and other patient-reported outcomes (PROs) in this population over time. [4, 5] Earlier studies reported worse QOL scores in older and female patients with SCD. [6–11] Moreover, low family income, low parental education, poor neighborhood, and other associated comorbidities were found to be associated with worse QOL scores in children with SCD in the United States. [9, 12, 13] However, the relationship between patients' characteristics and QOL scores is not well established in youth with SCD.

Hydroxyurea is a medication approved by the US Food and Drug Administration (FDA) for patients with SCD. There has been growing evidence to support the efficacy and the cost-effectiveness of using hydroxyurea in SCD. [14–18] Nevertheless, hydroxyurea adherence remains a challenge with adherence rates being suboptimal, on average 50-60%, which is even lower in youth with SCD. [19–22] Different barriers to hydroxyurea adherence have been reported in the literature, including fear of side effects; doubts about efficacy; lack of understanding of hydroxyurea benefits and side effects; lack of access or insurance coverage; forgetfulness; and the need for more frequent laboratory monitoring and clinic visits. [19, 20, 23–29] In patients with chronic health conditions, low medication adherence has been associated with worse health outcomes and increased healthcare utilization, [30–32] which was also seen in patients with SCD. [33] However, the relationship of patients' characteristics to hydroxyurea adherence and SCD-related clinical outcomes remains unclear in youth with SCD.

The objectives of this study were to assess the relationship between participants' characteristics and PROs, including QOL scores and hydroxyurea adherence rates, in youth with SCD, and to examine the relationship between participants' characteristics and SCD-related clinical outcomes in this population. We hypothesized that, among youth with SCD, older adolescent and young adults will have worse QOL scores, lower hydroxyurea adherence rates, and more frequent SCD-related complications. We also hypothesized that PROs scores, including QOL and hydroxyurea adherence, could vary by participant's gender, genotype, and other clinical characteristics.

## 2. Methods

### 2.1. Patient Enrollment

We approached all eligible patients before or after their scheduled clinic appointment in the comprehensive sickle cell clinic at Ann & Robert H. Lurie Children's Hospital of Chicago. Enrollment period extended from January 2015 to December 2015. Eligibility criteria included (1) age 12 to 22 years; (2) having a diagnosis of SCD (all genotypes) confirmed by hemoglobin electrophoresis; (3) being on a steady state dose of hydroxyurea with no dose changes for 2 months prior to study enrollment; and (4) being able to read and write in English. Exclusion criteria included (1) being on chronic pRBCs transfusion, either simple or exchange transfusions; (2) having a recent hospitalization or emergency room (ER) visit; (3) being concurrently treated for an acute pain episode at home; (4) having any physical or cognitive limitations that interfered with completing study procedures; and (5) having any inherited hemoglobin disorder other than SCD. The Institutional Review Board approved this study.

### 2.2. Study Outcomes

Endpoints and outcomes were planned based on our study objectives and a priori hypotheses. Different domains of QOL were evaluated using the Patient-Reported Outcomes Measurement Information System (PROMIS®) measures with computerized adaptive testing (CAT) functionality. PROMIS-CAT is a novel application of a comprehensive, item-response theory optimized item bank, which enables precise estimation of a PRO domain while simultaneously minimizing burden to participant. [34, 35] In CAT, items administered are selected based on informant's previous item responses, using a preset computerized algorithm based on individual item information functions. Therefore, the total number of items used in different PROMIS measures vary within and in-between patients. Pediatric and adult PROMIS® measures have been tested and validated in different patient populations, [36–39] including patients with SCD. [6, 40] Self-report PROMIS® measures included depression, anxiety, peer relationships, social isolation, fatigue, pain interference, upper extremity physical function, and mobility physical function. PROMIS® measures assessed patients' QOL based on the prior 7 days using a 5-point Likert scale. Higher PROMIS® scores indicated more severe or worse fatigue, pain interference, depression, or anxiety, while lower PROMIS® scores indicated more limited, impaired, or worse upper extremity physical function or mobility physical function. The mean T-score for PROMIS® measures has been established as 50 with a standard deviation of 10, based on pediatric and adult samples of the general population as well as patients with chronic health conditions. [36, 41]

©Modified Morisky Adherence Scale 8-items (©MMAS-8) was used to assess hydroxyurea adherence. ©MMAS-8 has been previously validated to assess medication adherence, and it includes seven dichotomous questions (Yes or No) and 1 multiple-choice question. [42, 43] Cumulative ©MMAS-8 score was calculated, and higher scores reflected higher adherence to hydroxyurea. Pretesting of ©MMAS-8 score was completed to confirm participants' understanding of different survey questions and response options.

A medical chart review was also conducted to assess (1) the number of SCD-related complications requiring hospitalization or ER visit in the year prior to study enrollment; (2) hydroxyurea indication, dose, and duration; and (3) prescribed long-acting narcotics (e.g., extended-release morphine), which was used to identify patients with chronic pain.

### 2.3. Statistical Analysis

All statistical analyses were planned based on our study objectives, a priori hypotheses, and clearly defined study outcomes. We reported descriptive statistics for categorical data as percentages and frequencies. Kruskal-Wallis test and Chi-square test were used to examine the associations between different variables. We used Spearman's correlations (r_s_) to evaluate the relationship between continuous variables. We analyzed the data to assess the potential relationship of participants' characteristics (i.e., age, gender, and having a history of chronic pain) to QOL scores, hydroxyurea adherence rates, and healthcare utilization (i.e., emergency room visits, hospitalizations, and length of hospital stay) in youth with SCD. Given our relatively small sample size, we did not conduct a multivariate modeling or regression analysis. Patients were categorized into two age groups: adolescents (12-17 years) and young adults (18-22 years), based on the data from the National Center on Birth Defects and Developmental Disabilities, Centers for Disease Control and Prevention. [44] Patients were also categorized based on the presence or absence of chronic pain. Chronic pain status was defined as daily pain for a minimum of 3 months and/or using long-acting narcotics (e.g., Morphine Sulphate Controlled Release or MS Contin) 2-3 times daily for a minimum of 3 months. Given the nature of our study being more hypothesis generating and descriptive in nature, a formal sample size calculation was not performed.* P*-value <0.05 was considered statistically significant. All tests were 2-sided. We conducted statistical analysis using StataCorp. 2013 (Stata Statistical Software. College Station, TX: StataCorp LP).

## 3. Results

### 3.1. Study Sample

**Figure 1:**
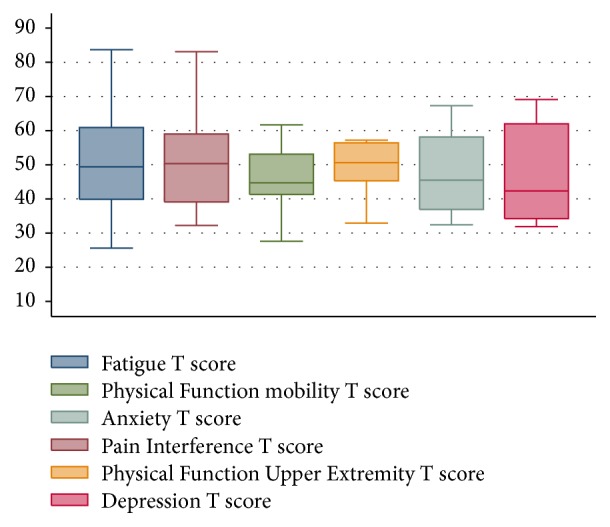
Participants' PROMIS® quality of life scores across different domains (T scores, median and interquartile range).

A total of 34 patients were approached in this cross-sectional study, and all agreed to participate and were enrolled (100% participation rate). All participants completed planned study procedures, including PROMIS® and ©MMAS-8 measures. Patients had a mean age of 14.8 years (SD 2.9), 41% were female, and the majority were African Americans (91%). Patients were prescribed hydroxyurea mainly for pain (53%) and about two-thirds of them (60%) had at least one hospitalization over the 12-month period preceding study enrollment. Patients had been on a mean hydroxyurea dose of 32.1 mg/kg/day (SD 4.9) for an average of 76.4 months in total (SD 50.3) and had been on a stable hydroxyurea for an average of 15.6 months (SD 8.8). Participants' characteristics are summarized in Table 1. All PROMIS® data were reported as T scores and participants' median (interquartile range [IQR]) scores were 49.4 (39.9, 60.9) for fatigue, 50.3 (39.1; 59) for pain, 44.7 (41.3; 53.1) for physical function mobility, 50.6 (45.3; 56.4) for physical function upper extremity, 45.5 (36.9; 58.1) for anxiety, and 42.3 (34.2; 62) for depression (Figure 1).

### 3.2. Participants Characteristics and QOL Domains

Participants' age correlated with their fatigue (r_s_=0.48;* P*=0.006), pain (r_s_=0.32;* P*=0.07), and anxiety (r_s_=0.33;* P*=0.06) scores, suggesting that older participants had more severe fatigue, pain, and anxiety. In support, young adults (age 18-22 years) had worse fatigue scores (median 60.7 versus 46.2,* P*=0.08), anxiety scores (median 57.3 versus 41.5, P=0.06), and depression scores (median 62.1 versus 37.5, P=0.14) compared to adolescents (age 12-17 years) (Table 2). Additionally, female participants had worse physical function mobility scores (median 43.5 versus 47.6,* P*=0.03) and fatigue scores (median 57.1 versus 45.3,* P*=0.11) compared to their male counterparts (Table 2).

Participants with chronic pain had worse pain (median 67.5 versus 49.9,* P*=0.04), upper extremity physical function (median 33.3 versus 50.7,* P*=0.05), physical function mobility (median 35.45 versus 46.2,* P*=0.09), anxiety (62.1 versus 42.3,* P*=0.05), and depression (64.35 versus 37.5,* P*=0.05) compared to those without chronic pain (Table 2). There were no significant differences in participants' PROMIS® QOL domain scores by genotype (data not shown). There were also no significant correlations between hydroxyurea dose or duration and any of the participants' QOL PROMIS® domain scores (data not shown). All PROMIS^®^ QOL domains scores were significantly intercorrelated (Table 3).

### 3.3. Participants Characteristics and Hydroxyurea Adherence

Using ©MMAS-8 scale, adherence levels could range from 0 to 8 with the higher number indicating better or more hydroxyurea adherence. Most participants (74%) reported low adherence to hydroxyurea using ©MMAS-8 with a mean (SD) score of 4.6 (1.7). Male participants reported significantly higher adherence to hydroxyurea using ©MMAS-8 (median 5.4 [IQR 3.8-6.5] versus 3.6 [IQR 2.5-5. 3],* P*=0.02) when compared to their female counterparts. There were no significant differences in participants' self-report of hydroxyurea adherence using ©MMAS-8 by age group (adolescents 12-17 years versus young adults 18-22 years) (*P*=0.89) or chronic pain status (*P*=0.88). There were also no significant correlation between participants' age and reported hydroxyurea adherence rates using ©MMAS-8 (*P*=0.91).

### 3.4. Participants Characteristics and Healthcare Utilization

Participants' healthcare utilization pattern varied in the year prior to study enrollment. Participants with chronic pain had significantly more frequent hospitalizations (median 8 [IQR 6-9] versus 1 [IQR 0-3],* P*=0.02) and emergency room visits (median 7 [IQR 5-9] versus 0 [IQR 1-4],* P*=0.04) as well as longer total length of inpatient hospital stays over a 12-month period (median 78 days [IQR 68-87] versus 3 days [0-19],* P*=0.01) compared to those without chronic pain. Female participants had more frequent hospitalizations (median 2.5 [IQR 1-4] versus 0 [IQR 0-2.5],* P*=0.09) and longer total length of inpatient hospital stays (median 14 days [IQR 1-24] versus 0 days [IQR 0-6],* P*=0.11) compared to male participants. Comparing adolescents (12-17 years) and young adults (18-22 years), there were no significant differences in number of hospitalizations (*P*=0.49), emergency room visits (*P*=0.25), or total inpatient length of stay (*P*=0.41).

## 4. Discussion

Our study contributes to the literature and the growing evidence on hydroxyurea adherence and QOL in youth with SCD. We found that older participants, female participants, and those with chronic pain had worse QOL scores, in particular pain, fatigue, physical function mobility, anxiety, and depression. We also found that females, relative to males, had significantly lower hydroxyurea adherence rates by self-report. In our cohort, participants with chronic pain had significantly more frequent hospitalizations and emergency room visits as well as longer total inpatient length of stay over a 12-month period when compared to those without chronic pain.

Our findings were consistent with data from other published studies that reported worse QOL scores in female patients and those of older age. [6–11, 45] In particular, Ingerski and colleagues reported significant differences in QOL scores related to age and gender across 8 different chronic health conditions (N=589), including SCD, cystic fibrosis, obesity, epilepsy, type 1 diabetes, postrenal transplantation, eosinophilic gastrointestinal disorder, and inflammatory bowel disease. [45] In our study, the association between older and worse QOL scores is likely related to the development of SCD-related complications over time in this population. [3, 4] Further, we found that female participants had worse QOL, compared their male counterparts, especially fatigue and physical function domains, which could be related to overall fitness and level of engagement with routine physical activity. Future research studies are needed to better understand the differences in participants' QOL scores as they relate to age and gender. Moreover, previous publications showed that low family income, poor neighborhood, low parental education, and associated comorbidities were found to be associated with worse QOL scores in children with SCD [9, 12, 13]; however, we did not evaluate these factors in our study. Although Dampier et al. showed worse QOL scores in patients with HbSS and HbSB^0^ thalassemia, [7] our findings were similar to data from other studies that failed to show differences in QOL based on patients' genotype. [6, 9] Although the differences in some of the QOL domains scores were not statistically significant between patient groups according to different characteristics, these T-score differences were clinically important, given that patients, parents, and physicians estimated T-score difference of 2-6 points to be clinically meaningful using PROMIS® measures. [46, 47] Our small sample size with inadequate power was likely related to our inability to detect statistically significant differences for these domains.

In patients with chronic health conditions, low medication adherence has been associated with worse health outcomes and increased healthcare utilization, [30–32] which was also seen in patients with SCD. [33] Hydroxyurea adherence has been a challenge for patients, parents, and providers, with the average adherence rate being 50-60%. [19–22] Reported barriers to hydroxyurea adherence in the literature included fear of side effects; doubts about efficacy; lack of understanding of hydroxyurea benefits and side effects; lack of access or insurance coverage; forgetfulness; and the need for more frequent laboratory monitoring and clinic visits. [19, 20, 23–26] In our study, we found that female participants had significantly lower hydroxyurea adherence rates by self-report when compared to males. However, there were no significant differences observed in participants' self-report of hydroxyurea adherence by age group or chronic pain status.

Patients with SCD have more frequent hospitalizations and emergency room visits compared to patients with other chronic health conditions. [48–53] Additionally, the youth with SCD were found to have increased healthcare utilization, in particular more frequent and longer hospitalizations, when compared to SCD patients in other age groups. [49–51, 53] Earlier publications showed increased healthcare utilization in SCD patients who were female,[49] had existing comorbidities or psychiatric disorders,[51, 54, 55] and lived in a remote area that was distant from a hospital. [48] In our study, chronic pain was significantly associated with more frequent hospitalizations and emergency room visits as well as longer total inpatient length of stay over a 12-month period.

Our study has some limitations worth discussing. First, this was a single-institution study with a relatively small sample size and the majority of participants being of African American descent, which may limit the generalizability of our findings, especially for SCD patients from the Middle East and Asia. Therefore, our study findings should be interpreted with caution. However, the data generated from this study are hypothesis generating and could inform a larger longitudinal study. Second, we used a self-report measure (©MMAS-8) to assess hydroxyurea adherence, which is subjective and limited by recall bias and social desirability. However, it has been used in earlier studies that included youth with SCD and other chronic illnesses and self-report of adherence correlated with other objective biomarkers of hydroxyurea use [19, 56]. Finally, we did not evaluate participants' perceived social support, which was highly correlated with adherence to therapy in previously published studies.

In conclusion, participants' characteristics were associated with PROs as well as SCD-related clinical outcomes. Participants' age positively correlated with their QOL scores, and females reported lower hydroxyurea adherence. Participants with chronic pain reported significant impairment in different QOL domains and had increased healthcare utilization. Therefore, future longitudinal studies are warranted to examine the relationship between participants' characteristics, QOL, hydroxyurea adherence, and SCD-related clinical outcomes. Furthermore, it is important to consider patients' characteristics in our efforts to develop and implement behavioral interventions for youth with SCD. There has been mounting evidence to support the efficacy of these interventions in improving adherence behavior among youth. [57–62] Therefore, given the ubiquitous access to mobile and personal technology, including among youth with SCD, [26] there is an opportunity to utilize behavioral intervention technologies to monitor and improve hydroxyurea adherence, QOL, and other important clinical outcomes in this population.

## Figures and Tables

**Table 1:** Participants' characteristics.

**Characteristics**	**Study cohort (N=34)**
Age (years), median (interquartile range)	13.5 (12 – 18)
Male, n (%)	20 (59)
Race/Ethnicity, n (%)	
African American	31 (91)
Hispanics or Latino	3 (9)
Genotype, n (%)	
HbSS	29 (85.3)
HbSC	3 (8.8)
HbSB^0^	2 (5.9)
Indication for hydroxyurea, n (%)	
Recurrent pain	18 (52.9)
Recurrent acute chest syndrome	2 (5.9)
Recurrent pain and acute chest syndrome	9 (26.5)
Others (poor growth, abnormal transcranial Doppler, or cerebrovascular accident)	5 (14.7)

**Table 2:** Participants' characteristics and PROMIS® quality of life domain scores.

**PROMIS® Domains, median (IQR)**	**Age**			**Gender**			**Chronic Pain** **∗**		
	**12–17 years**	**18-22 years**	***P***	**Male**	**Female**	***P***	**Yes**	**No**	***P***
**Fatigue**	46.2 (35; 57.1)	60.7 (51; 62.6)	0.08	45.3 (39.1; 54.1)	57.1 (48.8; 62)	0.11	61.5 (60.9; 62)	49 (39.9; 60)	0.17
**Pain **	49.2 (32.2; 59)	54.3 (51; 60.4)	0.34	50.1 (42; 59)	52.2 (32.2; 57.5)	0.5	67.5 (65.5; 69.5)	49.9 (39.1; 57.7)	**0.04**
**PF - UE**	50.2 (45.3; 57.2)	56.4 (44.2; 56.4)	0.89	50.7 (49.1; 57.2)	46.5 (44.3; 56.4)	0.15	33.3 (25.8; 40.8)	50.7 (46.4; 56.4)	**0.05**
**PF - mobility**	46.2 (41.3; 53.1)	44.1 (41.8; 54.1)	0.96	47.6 (43.8; 55.8)	43.5 (36.4; 47.3)	**0.03**	35.45 (27.6; 43.3)	46.2 (42.9; 53.1)	0.09
**Anxiety**	41.5 (32.4; 50.2)	57.3 (46.9; 61)	0.06	47.2 (36.9; 55.4)	41 (36.9; 59.7)	0.97	62.1 (60.9; 63.3)	42.3 (36.9; 55.4)	**0.05**
**Depression**	37.5 (31.9; 47.9)	62.1 (34.6; 63.1)	0.14	40.9 (34.6; 47.9)	42.3 (31.9; 62.2)	0.81	64.35 (62.2; 66.5)	37.5 (34.2; 52.1)	**0.05**

*P*-value < 0.05 was statistically significant and *p*-value of 0.05 was borderline significant (highlighted in bold).

All PROMIS® data were reported as T-scores.

IQR: interquartile range; PF: physical function; PROMIS: patient reported outcomes measurement information system; UE: upper extremities.

*∗* Chronic pain defined as daily pain for a minimum of 3 months and/or using long-acting narcotics (Morphine Sulphate Controlled Release or MS Contin) 2-3 times daily for a minimum of 3 months.

Higher PROMIS® scores indicated more severe or worse fatigue, pain interference, depression, or anxiety, while lower PROMIS® scores indicated more limited, impaired, or worse upper extremity physical function or mobility physical function.

We evaluated each PROMIS domain across patient groups separately with no adjustment for multiple testing.

**Table 3:** PROMIS® quality of life domains intercorrelations.

**PROMIS® Domains**	**Depression**	**Anxiety**	**PF-Mobility**	**PF-UE**	**Pain interference**
**Fatigue**	0.68 (*p*<0.0001)	0.54 (*p*=0.002)	-0.63 (*p*<0.001)	-0.39 (*p*=0.03)	0.69 (*p*<0.0001)
**Pain interference**	0.63 (*p*<0.001)	0.58 (*p*<0.001)	-0.40 (*p*=0.03)	-0.44 (*p*=0.01)	
**PF-UE**	-0.60 (*p*<0.001)	-0.47 (*p*=0.01)	0.62 (*p*<0.001)		
**PF-Mobility**	-0.55 (*p*=0.002)	-0.36 (*p*=0.04)			
**Anxiety**	0.83 (*p*<0.0001)				

*P*-value < 0.05 was statistically significant.

All PROMIS® data were reported as T-scores.

PF: physical function; PROMIS: patient reported outcomes measurement information system; UE: upper extremity.

Higher PROMIS® scores indicated more severe or worse fatigue, pain interference, depression, or anxiety, while lower PROMIS® scores indicated more limited, impaired or worse upper extremity physical function, or mobility physical function.

## Data Availability

The data generated from this study are not freely available because of institutional restrictions related to patient privacy as well as legal and ethical concerns.

## Abbreviation

CAT: Computerized adaptive testing
QOL: Quality of life
MMAS-8: Modified Morisky Adherence Scale 8-items
PROs: Patient-reported outcomes
PROMIS®: Patient-Reported Outcomes Measurement Information System
SCD: Sickle cell disease.
